# PivNG primers and probes set used in the cobas omni Utility Channel is a reliable supplemental test for detection of *Neisseria gonorrhoeae* in oropharyngeal, urogenital and rectal specimens collected in cobas PCR Media

**DOI:** 10.1136/sextrans-2022-055576

**Published:** 2023-04-28

**Authors:** Mark Hopkins, Rodney Arcenas, Xose Couto-Parada, Michael Lewinski, Merlin Njoya, Deva Perinpanathan, Rizwaan Sheriff, Avneet Hansra, Selena Singh

**Affiliations:** 1 Department of Infection and Immunity, Liverpool University Hospitals NHS Foundation Trust, Liverpool, UK; 2 Department of Infection and Immunity, Barts Health NHS Trust, London, UK; 3 Roche Molecular Systems Inc, Pleasanton, California, USA

**Keywords:** NEISSERIA GONORRHOEAE, Bacterial Infections, Molecular Diagnostic Techniques, Nucleic Acid Amplification Techniques, Polymerase Chain Reaction

## Abstract

**Objective:**

To evaluate the clinical performance of the novel PivNG primers and probes set (PivNG test) used in the cobas omni Utility Channel for supplemental testing of *Neisseria gonorrhoeae* (NG).

**Methods:**

Oropharyngeal, urogenital and rectal samples were self-collected during routine testing at Barts Health sexual health clinics, London, UK. Samples were tested by the cobas CT/NG test and PivNG cobas omni Utility Channel test on cobas 6800/8800 Systems. Supplemental testing was carried out with the Xpert CT/NG test. PivNG overall percent agreements, positive percent agreements (PPAs)/negative percent agreements (NPAs) and positive/negative predictive values were calculated for each sample type. Microscopy and/or culture data were included for a randomised subset of concordant/discordant results, and a composite reference standard (cobas CT/NG, Xpert CT/NG and culture results) adjusted for partial verification bias was used to determine PivNG PPA and NPA.

**Results:**

Of 447 evaluable samples with valid results from all three assays (cobas CT/NG, PivNG and Xpert CT/NG), 288 (64.4%) were NG-positive by both PivNG and cobas CT/NG; 117 (26.2%) were NG-negative in both tests; and 42 (9.4%) had discordant results (with NG-negative supplementary Xpert) CT/NG results in 40/42 instances). Of 19 PivNG/Xpert CT/NG-discordant samples, 11 were confirmed NG-positive by microscopy and/or culture results. PivNG PPA and NPA were 100% and 91% for oropharyngeal swabs, 100% and 100% for vaginal swabs, 100% and 100% for male urine samples, and 100% and 97% for rectal swabs, respectively, compared with the partially adjusted composite reference standard.

**Conclusions:**

PivNG is a reliable supplementary test with high sensitivity for confirming NG infection when used in conjunction with the cobas CT/NG test and samples collected in cobas PCR Media. Moreover, the PivNG test offers a convenient, high-throughput solution for supplemental NG testing of various sample types, with the potential to reduce the number of indeterminate reports.

## Introduction

*Neisseria gonorrhoeae* (NG) is the second most common bacterial STI,^1 2^ causing an estimated 82 million new cases of gonorrhoea worldwide in 2020.^1^ Transmission is by direct mucosal contact and can lead to symptomatic or asymptomatic infections in the urethra, endocervix, rectum and pharynx.^2^

NG is diagnosed by detection using a sample collected from the infected site with nucleic acid amplification tests (NAATs), culture or direct microscopy.^2^ NAATs are more sensitive and easier to perform and provide results faster than culture;^2 3^ however, cross-reactivity can occur due to commensal *Neisseria* species at the same sites.^3^ Therefore, UK screening guidelines recommend a supplementary NAAT if the positive predictive value (PPV) of the initial test is <90% for the population tested.^3 4^

The cobas CT/NG test for use on cobas 6800/8800 Systems (Roche Molecular Systems Inc., USA) is a NAAT for direct detection of *Chlamydia trachomatis* (CT) and/or NG DNA from various specimens, including extragenital sources.^5^ The cobas CT/NG test targets a highly conserved direct repeat (DR9) region of the NG genome.

The cobas 6800/8800 Systems are fully automated and high throughput, designed primarily for running commercial/CE-marked PCR assays, and they have open-channel functionality capable of performing laboratory-developed tests on the cobas omni Utility Channel. A novel alternative primers and probes set targeting the NG-specific *pilin inversion* (*piv*) gene was provided by Integrated DNA Technologies for use on this channel.^6^ Using the cobas omni Utility Channel for supplementary testing removes the need for additional laboratory equipment.

The objective of this study was to evaluate the clinical performance of the PivNG primers and probes set (PivNG test) for supplemental testing of NG in oropharyngeal, urogenital and rectal samples.

## Methods

### Study design and specimen collection

Oropharyngeal, vaginal, male urine and rectal specimens were self-collected in cobas PCR Media by patients attending routine testing for STIs at Barts Health sexual health clinics, London, UK. Patients unwilling or unable to self-collect samples were excluded. Samples were obtained and tested from November 2019 to June 2020. Samples were initially tested using the cobas CT/NG test as part of routine screening. Only samples with sufficient surplus volume for PivNG and Xpert CT/NG test (Cepheid) supplemental testing were included in the evaluation.

### Sample testing

Specimens were tested for NG with the CE-IVD cobas CT/NG test and also the PivNG cobas omni Utility Channel test on the cobas 6800/8800 Systems, following the manufacturer’s recommended instructions for use. Supplemental testing was also carried out using the Xpert CT/NG test by adding 300 µL of sample collected in cobas PCR Media to the Cepheid cartridge. NG culture and microscopy results were used, if available, for concordant/discordant sample analysis.

Although all samples were self-collected by the patient, neither the cobas CT/NG test nor the Xpert CT/NG test is licensed for use with patient self-collected oral or anorectal swabs. Self-collected vaginal swabs are on-label for both assays. Self-collected extra-genital swabs were validated locally by BARTS Health NHS Trust where samples were co-collected in Aptima and cobas media to compare performance with the Hologic Panther.

### Data analysis

All analyses were performed using SAS/STAT software V.9.4. The overall percent agreement (OPA), positive percent agreement (PPA), negative percent agreement (NPA), PPV and negative predictive value of the PivNG test with respect to specimen type and compared with the cobas CT/NG test were calculated, along with corresponding two-sided 95% CIs using Wilson’s score method. A sample size of 60 ensured a precision level (half-width of 95% CI) of 3%–8%, assuming agreement of 90–100%. Discordant analysis evaluated results of NAATs, microscopy and culture, as explained in the online supplemental methods. Invalid/failed tests were excluded from the data analyses. Analytical sensitivities of the PivNG and Xpert CT/NG tests were determined, as explained in the online supplemental methods. Analytical specificity of the PivNG test was determined using a panel of 20 non-gonococcal isolates. NPA was determined by (number of PivNG-negative results/total number of non-gonococcal isolates)×100, and CIs were calculated using the Clopper-Pearson exact binomial CI method.

10.1136/sextrans-2022-055576.supp1 Supplementary data

## Results

### Characteristics of clinical samples

Overall, 447 evaluable samples were obtained from 377 patients (28.9% (109) female and 70.3% (265) male, 0.8% (3) unknown), with a mean age of 32.5±10.8 years. Of the 377 patients who contributed evaluable samples, 83.6% (315) provided one type; 14.3% (54) provided two types; and 2.1% (8) provided three types.

### Performance of the PivNG test for secondary detection of NG infection

The OPA was 90.6% between PivNG and cobas CT/NG tests, with full concordance for 288 of 447 (64.4%) samples, and 117 of 447 (26.2%) samples were NG-negative with both tests (table 1). Discordant PivNG and cobas CT/NG results were recorded for 42 (9.4%) samples, of which 40 were NG-negative using the supplementary Xpert CT/NG test. The majority (27 of 40) were oropharyngeal swabs (table 1). Culture and microscopy results were unavailable from this collection site. Microscopy and/or culture NG results were included for a randomised subset of concordant/discordant NAAT results (online supplemental table S1). A composite reference standard was created using the cobas CT/NG, Xpert CT/NG and culture results. When adjusted for partial verification bias and compared with the composite reference standard, PivNG gave a PPA/NPA of 100%/91% for oropharyngeal swabs, 100%/100% for vaginal swabs, 100%/100% for male urine samples and 100%/97% for rectal swabs, respectively (online supplemental table S2).

**Table 1:** Diagnostic performance for detection of NG DNA using PivNG compared with the cobas CT/NG test and with reactive samples retested using Xpert CT/NG as a supplementary NAAT

	Positive or negative samples by assay (n)				Performance		
	cobas NG (+) and PivNG (+)	cobas NG (+) and PivNG (−)	cobas NG (-) and PivNG (+)	cobas NG (−) and PivNG (−)	OPA % (95% CI)	PPA% (95% CI), NPA % (95% CI)	PPV % (95% CI), NPV % (95% CI)
All samples*	**288**	**40**	**2**	**117**	90.6 (87.5 to 93.0)	87.8 (83.8 to 90.9), 98.3 (94.1 to 99.5)	99.3 (97.5 to 99.8), 74.5 (67.2 to 80.7)
Of which Xpert NG+	269	0	0				
Xpert NG−	19	40	2				
**Oropharyngeal swab samples**	**87**	**27**	**2**	**38**	81.2 (74.3 to 86.6)	76.3 (67.7 to 83.2), 95.0 (83.5 to 98.6)	97.8 (92.2 to 99.4), 58.5 (46.3 to 69.6)
Of which Xpert NG+	74	0	0				
Xpert NG−	13	27	2				
**Vaginal swab samples**	**62**	**7**	**0**	**28**	92.8 (85.9 to 96.5)	89.9 (80.5 to 95.0), 100 (87.9 to 100.0)	100 (94.2 to 100.0), 80.0 (64.1 to 90.0)
Of which Xpert NG+	60	0	0				
Xpert NG−	2	7	0				
**Male urine samples**	**57**	**2**	**0**	**25**	97.6 (91.7 to 99.3)	96.6 (88.5 to 99.1), 100 (86.7 to 100.0)	100 (93.7 to 100.0), 92.6 (76.6 to 97.9)
Of which Xpert NG+	56	0	0				
Xpert NG−	1	2	0				
**Rectal swab samples**	**82**	**4**	**0**	**26**	96.4 (91.2 to 98.6)	95.3 (88.6 to 98.2), 100 (87.1 to 100.0)	100 (95.5 to 100.0), 86.7 (70.3 to 94.7)
Of which Xpert NG +	79	0	0				
Xpert NG−	3	4	0				

Main analysis (cobas NG vs PivNG) values shown in bold

*Some patients provided more than one sample type.

+, positive; −, negative; CT, *Chlamydia trachomatis*; NAAT, nucleic acid amplification test; NG, *Neisseria gonorrhoeae*; NPA, negative percent agreement; NPV, negative predicted value; OPA, overall percent agreement; Piv, pilin inversion; PPA, positive percent agreement; PPV, positive predicted value.

When determining analytical performance, PivNG had a lower limit of detection for cobas NG-positive samples compared with Xpert CT/NG (online supplemental table S3) but retained high specificity when tested with non-gonococcal isolates (online supplemental table S4).

### Supplementary Xpert CT/NG testing of samples in cobas PCR Media

NG-negative Xpert CT/NG results were also recorded for 19 samples with concordant NG-positive cobas CT/NG/PivNG results (13 oropharyngeal, 2 vaginal, 1 male urine and 3 rectal samples) (table 1). Oropharyngeal sites were not sampled for culture; however, urethral (n=10) or rectal (n=1) NG infection was confirmed by culture and/or microscopy in 11 of 19 of these patients (data not shown), indicating Xpert CT/NG false negativity. However, overall concordance between PivNG and Xpert CT/NG as secondary tests was good (40 negative and 269 positive) (table 1).

AmpliRun Total CT/NG/TV/MGE control (Vircell) material diluted in cobas PCR Media and molecular grade water indicated that cobas PCR Media was not inhibitory to Xpert CT/NG, but cobas CT/NG and PivNG reported positive results at lower concentrations using this protocol (online supplemental table S5).

## Discussion

Previous studies have established the performance of the cobas CT/NG test for detecting NG infection.^5 7^ This study evaluated clinical performance of the PivNG test as a supplementary test using the cobas 6800/8800 Systems in conjunction with the cobas CT/NG test. The PivNG test performed well with all specimen types and appeared a reliable secondary test for confirming NG infection using samples collected in cobas PCR Media. Improved sensitivity of PivNG compared with the Xpert CT/NG test in this setting was also supported by direct comparison of analytical performance.

Lower agreement between cobas CT/NG and PivNG tests was observed with oropharyngeal swabs compared with other sample types. Unfortunately, culture and microscopy results were unavailable for this subset of samples in our discordant analysis due to local practice, where the decision to sample different anatomical sites for culture/microscopy is guided by risk and symptoms at those sites during clinical presentation. Perry *et al* reported a lower PPV for detecting NG in oropharyngeal samples (88.6%) than rectal (96.4%) or urogenital (96.0%) samples.^8^ These data, along with the data from our study, highlight that rectal and urogenital samples may not require supplemental testing, but oropharyngeal samples should undergo secondary testing, in line with the UK guidelines.^3 4^ This is consistent with the literature regarding the presence of commensal *Neisseria*, particularly in the pharynx,^3^ and genetic exchange of NG DNA to these species, which can result in false positivity due to cross-reactivity with non-NG species when performing NAATs.^9^ While our results suggest good performance of the cobas CT/NG test and PivNG test with specimens collected in cobas PCR Media, we suggest that cobas NG-positive but secondary PivNG-negative samples are reported as equivocal or indeterminate, with a repeat specimen request and subsequent PPV audit to inform a local reporting algorithm. The concern over cross-reactivity in extragenital samples needs to be balanced with the NAAT cycle threshold value, as samples with high values have an increased likelihood of non-confirmable results.^10^ Using highly sensitive screening, as well as reporting discordant samples as equivocal or indeterminate, allows therapy decisions to be influenced by pretest probability.

Our study has some limitations. The sample size was relatively small and varied across sample types. Determining PivNG performance in a larger sample set, with similar sized groups, would be beneficial to confirm results and enable clear comparisons across sample types. Analytical performance was evaluated only for the PivNG and Xpert CT/NG tests, not the cobas CT/NG test; the latter may have provided more clarity when evaluating discordant samples. Finally, all samples were routinely collected in cobas PCR Media, which is an off-label use for the Xpert CT/NG test and could affect performance. Brief evaluation of Xpert CT/NG analytical sensitivity with this protocol showed no evidence of inhibition, but the limit of detection appeared suboptimal. The collection of specimens in a single edia type is a real-world issue for laboratories when selecting supplementary tests. Our data show supplementary assay choice could influence the number of equivocal reports issued in settings where cobas PCR Media is used.

In conclusion, this is the first study to show the clinical performance of the PivNG test on the cobas omni Utility Channel. The performance of PivNG was good in conjunction with the cobas CT/NG test and could result in fewer equivocal reports being issued to clinic. This open-channel assay offers a convenient and high-throughput solution for supplemental NG testing of various sample types.
