# Supplementary material for: PivNG primers and probes set used in the cobas omni Utility Channel is a reliable supplemental test for detection of Neisseria gonorrhoeae in oropharyngeal, urogenital and rectal specimens collected in cobas PCR Media
Source: Sex Transm Infect. 2023 Apr 28;99(6):416–9. doi: 10.1136/sextrans-2022-055576 (PMC10447364; doi:10.1136/sextrans-2022-055576)
Supplement: Supplementary data [file sextrans-2022-055576supp001.pdf]

Supplementary Material

The PivNG primers and probes set used in the cobas omni Utility Channel is a reliable supplemental test for detection of *Neisseria gonorrhoeae* in oropharyngeal, urogenital and rectal specimens collected in cobas PCR Media

Mark Hopkins, Rodney Arcenas, Xose Couto-Parada, Michael Lewinski, Merlin Njoya, Deva Perinpanathan, Rizwaan Sheriff, Avneet Hansra, Selena Singh

Supplementary Methods

Composite reference standard analysis of subset of discordant and concordant NAAT NG results with available microscopy and/or culture results

Given that microscopy and/or culture data were included for a randomised subset of samples with cobas® CT/NG and Xpert® CT/NG concordant and discordant NG results, the composite reference standard was partially verified. The randomised selection contained a total of 111 samples covering 59 discordant samples and a subset of concordant positive (36/269), concordant negative (5/40) and missing cobas/invalid Xpert (11/85) results. Of these, culture/microscopy results were available for 8 rectal (2 negative, 6 positive), 8 oropharyngeal (6 negative, 2 positive), 12 male urine (1 negative, 11 positive) and 6 vaginal (4 negative, 2 positive) samples. The Begg and Greenes' correction method[1] was used to calculate partial verification bias-adjusted estimates of PPA and NPA on the entire dataset (453 samples with valid PivNG results), with the assumption that the undetermined composite reference standard status was at random. Of this dataset, 6 samples were not evaluable (2 were missing both cobas CT/NG and Xpert CT/NG results and 4 had invalid Xpert CT/NG results) when determining the performance of PivNG compared with cobas CT/NG and Xpert CT/NG (Table 1; 447 samples).

Analytical performance

Analytical sensitivity, defined as the lowest concentration where ≥95% of samples tested give positive results, was calculated using a probit curve fit to the lowest panel members. A panel per sample type with 25 replicates per level were tested in two specimen matrices over 5 days. Levels evaluated were 0.01, 0.03, 0.1, 0.3, 1 and 3 colony-forming units (CFUs)/mL for the swab matrix, and 0.005, 0.015, 0.05, 0.15, 0.5 and 1.5 CFUs/mL for the

Hopkins M, *et al. Sex Transm Infect* 2023; 99:416–419. doi: 10.1136/sextrans-2022-055576

urine matrix. Swab samples were collected in cobas PCR Media spiked with human colorectal carcinoma HCT-15 cells, and urine samples were collected in upper urinary tract matrix.

Limit of detection analysis of assays using a third-party control diluted in cobas PCR Media or molecular grade water

Amplirun® Total CT/NG/TV/MGE control material (Vircell) was serially diluted using either cobas PCR Media or molecular grade water to six dilution points with nominal concentrations of 111, 36, 8, 4, 1, 0.1 copies/mL in each media, spanning the limit of detection of the Xpert CT/NG test. The panel of 12 samples (six concentrations in each media) were tested in triplicate using cobas CT/NG, PivNG and Xpert CT/NG assays and results compared.

Reference

1. Arifin WN, Yusof UK. Correcting for partial verification bias in diagnostic accuracy studies: A tutorial using R. *Stat Med* 2022;41:1709–27.

Supplementary Table S1. Frequency of available microscopy and culture NG results, with PivNG results stratified by concordant and discordant NG results from cobas CT/NG and Xpert CT/NG

Specimen site	cobas/Xpert NG results	Microscopy and culture NG result	PivNG result	Frequency
**Oropharyngeal swab**	Concordant negative	Not available	NG negative	38
	Concordant negative	Not available	NG positive	2
	Concordant positive	Negative	NG positive	1
	Concordant positive	Not available	NG positive	72
	Concordant positive	Positive	NG positive	1
	Discordant positive/negative	Negative	NG negative	1
	Discordant positive/negative	Negative	NG positive	4
	Discordant positive/negative	Not available	NG negative	26
	Discordant positive/negative	Not available	NG positive	8
	Discordant positive/negative	Positive	NG positive	1
	Missing cobas/invalid Xpert	Not available	NG negative	2
**Vaginal swab**	Concordant positive	Negative	NG positive	2
	Concordant positive	Not available	NG positive	56
	Concordant positive	Positive	NG positive	2
	Discordant positive/negative	Negative	NG negative	1
	Discordant positive/negative	Negative	NG positive	1
	Discordant positive/negative	Not available	NG negative	6
	Discordant positive/negative	Not available	NG positive	1
	Missing cobas/invalid Xpert	Not available	NG negative	31
**Male urine sample**	Concordant positive	Not available	NG positive	45
	Concordant positive	Positive	NG positive	11
	Discordant positive/negative	Negative	NG negative	1

Hopkins M, *et al. Sex Transm Infect* 2023; 99:416–419. doi: 10.1136/sextrans-2022-055576

	Discordant positive/negative	Not available	NG negative	1
	Discordant positive/negative	Not available	NG positive	1
	Missing cobas/invalid Xpert	Not available	NG negative	25
**Rectal swab**	Concordant positive	Not available	NG positive	73
	Concordant positive	Positive	NG positive	6
	Discordant positive/negative	Negative	NG negative	1
	Discordant positive/negative	Negative	NG positive	1
	Discordant positive/negative	Not available	NG negative	3
	Discordant positive/negative	Not available	NG positive	2
	Missing cobas/invalid Xpert	Not available	NG negative	26
	Missing cobas/invalid Xpert	Not available	NG positive	1

CT, *Chlamydia trachomatis*; NG, *Neisseria gonorrhoeae*; Piv, pilin inversion

Hopkins M, *et al. Sex Transm Infect* 2023; 99:416–419. doi: 10.1136/sextrans-2022-055576

Supplementary Table S2. PivNG PPA and NPA with a composite reference standard adjusted for partial verification bias using Begg and Greenes' method

Specimen site	PivNG	Composite reference standard (cobas and Xpert and culture)				PPA (95% CI)	NPA (95% CI)
		**Positive**	**Negative**	**Undetermined**	**Total**		
**Oropharyngeal swab**	Positive	75	6	8	89	100% (99.96, 100.00)	91.04% (84.25, 97.83)
	Negative	0	39	28	67		
	Total	75	45	36	156		
**Vaginal swab**	Positive	60	1	1	62	100% (99.07, 100.00)	100% (99.93, 100.00)
	Negative	0	1	37	38		
	Total	60	2	38	100		
**Male urine sample**	Positive	56	0	1	57	100% (99.07, 100.00)	100% (99.93, 100.00)
	Negative	0	1	26	27		
	Total	56	1	27	84		
**Rectal swab**	Positive	79	1	3	83	100% (99.28, 100.00)	96.66% (90.22, 100.00)
	Negative	0	1	29	30		
	Total	79	2	32	113		

CI, confidence interval; NG, *Neisseria gonorrhoeae*; NPA, negative percent agreement; Piv, pilin inversion; PPA, positive percent agreement

Hopkins M, *et al. Sex Transm Infect* 2023; 99:416–419. doi: 10.1136/sextrans-2022-055576

Supplementary Table S3. Summary limit of detection estimates for cobas NG-positive samples using PivNG compared with Xpert CT/NG

Matrix	Assay	LOD probit estimate (CFU/mL)	95% CI
Swab	PivNG	0.043	0.026, 0.199
	Xpert NG	0.326	0.162, 1.671
Urine	PivNG	0.018	0.011, 0.121
	Xpert NG	0.451	0.186, 3.136

CFU, colony-forming unit; CI, confidence interval; LOD, limit of detection; NG, *Neisseria gonorrhoeae*; Piv, pilin inversion

Supplementary Table S4. NPA between a specificity panel of non-gonococcal isolates and PivNG-negative results

Expected result	Number of non-gonococcal isolates*	PivNG (+) results	PivNG (-) results	NPA (%)	95% exact CI
Negative	20	0	20	100	83.2, 100.0

*Non-gonococcal *Neisseria* isolates evaluated were: *N. cinerea*, *N. dentrificans*, *N. elongata subsp. elongata*, *N. elongata subsp. niroreducans*, *N. flava*, *N. flavescens*, *N. subflava*, *N. lactamica*, *N. macacae*, *N. meningitidis Serogroup A*, *N. meningitidis Serogroup B*, *N. meningitidis Serogroup C*, *N. meningitidis Serogroup D*, *N. meningitidis Serogroup W135*, *N. meningitidis Serogroup Y*, *N. mucosa*, *N. perflava*, *N. polysaccharea*, *N. sicca* and *N. subflava*

+, positive; -, negative; CI, confidence interval; NG, *Neisseria gonorrhoeae*; NPA, negative percent agreement; Piv, pilin inversion

Hopkins M, *et al. Sex Transm Infect* 2023; 99:416–419. doi: 10.1136/sextrans-2022-055576

Supplementary Table S5. Performance of Xpert CT/NG with Vircell control material diluted to six nominal concentrations using cobas PCR Media or molecular grade water

Vircell control diluted in cobas PCR Media					Vircell control diluted in molecular grade water				
	Xpert NG*		cobas NG	PivNG		Xpert NG		cobas NG	PivNG
NG copies/mL	NG2 Ct	NG4 Ct	NG Ct	PivNG Ct	NG copies/mL	NG2 Ct	NG4 Ct	NG Ct	PivNG Ct
111	32.4	32.7	34.22	30.4	111	31.9	31.8	34.14	30.6
111	32.5	32.6	34.28	30.5	111	31.9	31.5	33.84	30.5
111	32.6	32.2	33.84	30.3	111	32.3	32.1	34.75	30.6
36	35	34.8	36.53	32.2	36	34.6	33.5	35.67	32.2
36	33.9	34.1	35.92	31.8	36	34.4	33.7	36.71	32
36	34.4	33.5	36.02	32.4	36	35	34.4	35.27	32.3
8	Not detected	36.1	36.69	35	8	37.4	Not detected	39.08	34.3
8	Not detected	37.7	37.5	34.5	8	35.7	39.4	37.8	34.3
8	37.2	37.2	39.62	34.3	8	Not detected	37.1	38.28	35
4	Not detected	37.4	40.06	35.7	4	36.1	37.9	39.27	35.1
4	37.7	37.4	38.8	36.5	4	Not detected	37.9	41.67	35.2
4	36.7	Not detected	40.7	35.3	4	Not detected	35.9	Not detected	35.5
1	Not detected	Not detected	Not detected	Not detected	1	Not detected	Not detected	39.15	Not detected
1	Not detected	Not detected	39.83	35.8	1	Not detected	Not detected	Not detected	37.4
1	Not detected	Not detected	Not detected	Not detected	1	Not detected	Not detected	Not detected	Not detected
0.1	Not detected	Not detected	Not detected	Not detected	0.1	Not detected	42	Not detected	Not detected
0.1	Not detected	Not detected	Not detected	Not detected	0.1	Not detected	Not detected	Not detected	Not detected
0.1	Not detected	Not detected	Not detected	Not detected	0.1	Not detected	Not detected	Not detected	Not detected

*Xpert CT/NG requires both NG targets (designated by Cepheid as NG2 and NG4) to be detected to assign a positive result

CT, *Chlamydia trachomatis*; Ct, cycle threshold; NG, *Neisseria gonorrhoeae*; Piv, pilin inversion
